# Exergoeconomic and enviroeconomic evaluations of conventional solar still using PCM and electric heater powered by solar energy: an experimental study

**DOI:** 10.1007/s11356-023-26761-4

**Published:** 2023-04-25

**Authors:** Eslam Ahmed Abdel-Aziz, Tamer M. Mansour, Mohamed M. Khairat Dawood, Tamer M. Ismail, Khaled Ramzy

**Affiliations:** 1 Department of Mechanical Engineering, Sinai University, Sinai, Egypt; 2 Department of Mechanical Engineering, Suez Canal University, Ismailia, Egypt

**Keywords:** Solar energy, Desalination, Solar still, Performance, Freshwater production, PCM, Solar heater

## Abstract

Solar stills are used in distant and arid areas to convert brackish or salty water into potable water fit for human use in a simple, affordable, and effective manner. Even when PCM materials are used, typical solar systems still have minimal production per day. In this study, experimental tests were carried out in order to increase the performance of a single-slope solar still combined with PCM material (paraffin wax) and a solar-powered electric heater. Two identical single-slope solar stills were designed, fabricated, and tested under the same climatic conditions during the summer and spring seasons of 2021 in Al-Arish, Egypt. The first is a conventional solar still (CVSS), and the other is also a conventional still but with PCM and an electric heater (CVSSWPCM). Several parameters were measured during the experiments, including sun intensity, meteorological aspects, cumulative freshwater production, average glass, and water temperatures and PCM temperature. The improved solar still was evaluated at different operating temperatures and was compared to the conventional traditional one. There were four cases studied: one case without a heater (paraffin wax only) and three other cases with a heater operating at 58 °C, 60 °C, and 65 °C, respectively. The experimental results revealed that activating the heater inside the paraffin wax increased daily production (i) in the spring by 2.38, 2.66, and 3.1 times and (ii) and in the summer by 2.2, 2.39, and 2.67 times at the three above-mentioned temperatures respectively (when compared to the traditional still). In addition, the maximum rate of daily freshwater production was achieved at paraffin wax temperature of 65 °C in both spring and summer (Case 5). Finally, the economic evaluation of the modified solar still was carried out according to cost per litre. The modified solar still with a heater operating at 65 °C has a higher exergoeconomic value than the traditional one. The maximum CO_2_ mitigation in cases 1 and 5 was approximately 28 tons and 160 tons, respectively.

## Introduction

Energy and water scarcity are the two major global challenges that affect every country’s economic development. By the year of 2025, about 33% of the world’s population will be living in water-stressed countries (Zhang et al. 2018). Beside the few resources of potable and drinkable water, the crucial problem facing the water use is the pollution which requires an energy-consuming distillation process. The distillation process requires about 0.71 kWh of energy to generate 1 m^3^ of fresh water; 1 ton of oil at least must be fired to produce 20 tons of distilled water (Reddy and Sharon 2016). Solar energy is a clean viable alternative that is superior to conventional fuels which pollute the environment. Solar distillation is one of the best solutions for meeting the needs for providing drinkable water (Bhaisare et al. 2019). There are two main types of solar stills: (i) passive solar stills which use the solar power as the main source to derive the distillation process and (ii) active solar stills which use a secondary power source besides the solar power to derive the distillation process. Solar still, which works like the cycle of rain in nature, consists of a blacked basin under a transparent cover. Solar radiation heats the saline water in the basin, allowing it to evaporate. Moisture rises, condenses on the cover glass, and drips into a collecting trough, getting rid of salts, minerals, and other impurities. The freshwater production of passive solar still ranges between 2 and 5 l/m^2^ day (Kabeel and El-Agouz 2011), making this system highly uneconomical and inefficient. In addition, one of the main problems of solar stills is their lack of freshwater production at night due to the absence of the sunlight, a thing that makes them unable to produce potable water at night. The solar still’s efficiency can be enhanced to 60% by storing the energy available during peak hours by using phase change material (PCM) acting as a heat source for saline water during the night hours (Saikrishnan and Karthikeyan 2016). One of the most important advantages of coupling PCM with solar stills is that PCM stores more heat (5–14 times), when compared to the sensible heat storage material (Dinker et al. 2017). PCMs are classified into three main types: the first is the organic PCM, such as paraffin wax; the second is the inorganic PCM, such as salt-hydrate; and the third is the eutectic PCM, such as organic-organic and organic–inorganic PCM types. When the temperature applications are low (10–80 °C), as the case in all solar still applications, a PCM with a lower melting temperature (such as paraffin wax) is preferable as it can help to maintain a lower operating temperature (Hu et al. 2014; Andrassy and Szantho 2019).

Furthermore, paraffin wax is the preferred choice due to its widespread availability, low cost, ease of recycling, slight volume changing during phase change, good phase equilibrium, and low vapour pressure and melting temperature. PCMs in general, and paraffin wax in particular, have significant drawbacks such as poor thermal conductivity which causes the heat transfer rate to decrease during charging/discharging cycles (Boukani et al. 2018). Various research projects are being carried out in order to improve the freshwater production of solar stills, both passive and active, and to overcome the problem of paraffin wax’s lower thermal conductivity. Omara et al. (2020) studied different types of PCMs and all their properties, provided a detailed review of the usage of PCMs in most passive and active solar still types. According to their findings, organic PCMs (such as paraffin wax) were commonly employed in numerous investigations, but relatively few studies investigated the impacts of inorganic and eutectic PCMs. Also, their study indicated that the freshwater production of solar stills increased along with an increasing PCM mass and a decreasing saline water mass. In addition, adding only PCM or PCM with additions such as nanoparticles to the passive solar still types improves the freshwater production up to 127%. A review of the effects of using phase change materials on solar stills performance was conducted by Katekar and Deshmukh (2020). Different types of active and passive solar stills loaded with all types of phase change materials (paraffin wax, lauric acid, bitumen, stearic acid, palmitic acid, capric acid, and meristic acid) had been compared. The study concluded that the paraffin wax was the most appropriate PCM for passive as well as active solar stills because of its higher latent heat of fusion when compared to all the other PCMs used in solar stills. The results concluded that for a single basin passive solar still, paraffin wax gave the best enhancement in freshwater production, energy, and exergy efficiency by 180%, 67.2%, and 40%, respectively. While the highest freshwater production was about 307.54% using the paraffin wax in the active solar still. Kabeel et al. (2018) conducted a theoretical comparison for the behaviour of various PCMs (inorganic and organic) on the conventional still. The results concluded that inorganic and organic PCMs had the highest yield and the lowest cost. It is also shown that the PCM thickness has no effect on the output. Naim and Abd El Kawi (2003) used a mixture of paraffin wax, paraffin oil, and water as a PCM in a multiple-basin solar still. The results showed that the maximum freshwater production was about 4.53 l/m^2^ in 6 h for salty water flow rate of 40 ml/min with a 36.2% energy efficiency. Ansari et al. (2013) studied the desalination of brackish water numerically using a passive solar still with three different PCMs with different melting temperatures. The results showed that the selection of PCM greatly depended on the maximum temperature of brackish water in the basin. Also, a significant improvement in the freshwater production for the solar still was detected along with an increasing melting point of the PCM. Kumar et al. (2015) studied the use of paraffin wax as a latent heat storage medium in a passive single-basin double-slope solar still. The results showed that an overall 61% gain was obtained with PCM usage. The freshwater production of single-slope passive solar still was enhanced experimentally by adding paraffin wax as a heat storage medium by Kabeel and Abdelgaied (2016). Two solar stills were constructed and tested to compare their freshwater production. The first one was a solar still with paraffin wax, while the other was a conventional one. The results showed that the accumulated freshwater production per day of the solar still with paraffin wax was 67.18% higher than that of the still without paraffin wax. The effect of impregnating different types of nanoparticles in paraffin wax (NPCM) was studied experimentally by Rufuss et al. (2018). Three different types of NPCM were added to three passive solar stills with the same dimensions and compared to another passive solar still with PCM. The hourly freshwater production of the solar still with PCM was 3.92 l/m^2^ day, the solar still with NPCM-1 was 4.94 l/m^2^ day, the solar still with NPCM-2 was 5.28 l/m^2^ day, while the solar still with NPCM-3 was 3.66 l/m^2^ day. An experimental study was performed by Yousef and Hassan (2019) on a modified single-slope solar still and was then compared with the conventional one with the same dimensions. Four cases were studied: conventional solar still, still with PCM (paraffin wax), still with PCM and pin fin heat sinks embedded in the PCM (PCM-PF), and still with PCM and black steel mesh fibres in the basin (PCM-SWF). Compared to the production of the conventional still, the total accumulated freshwater production of the still with PCM was 9.5% greater, the still with PCM-PF 16.8%, and the still with PCM-SWF 13% per day. Kabeel et al. (2019) experimentally tested and compared a solar still with an internal reflector with a mixture of black gravel and Paraffin wax. This composite material with PCM was used to enhance the freshwater production. The results indicated that the freshwater production in the case of using the said composite was 3.27 l/m^2^ with augmentation by 37.55% higher than the case of using paraffin wax only. Khalilmoghadam et al. (2020) experimentally tested a passive solar still that included a PCM unit and a pulsing heat pipe. The results concluded that the system efficiency increased from 23.7% in the conventional solar still to 48.5% in the modified system where the cost per litre was 0.0093 $/l/m^2^. Energy and exergy methodologies for a passive solar still with and without paraffin wax as PCM were tested in the summer and the winter by Yousef and Hassan (2020). The experimental results showed that adding paraffin wax to the passive solar still improved energy by 10% and energy savings by 3% per year. When compared the conventional still, the results showed that the total freshwater production of the trays still was improved by 57% when using reflectors, by 14% when using CuO nanoparticles in paint, by 71% when using reflectors and nano-coating, and by 108% when using a collection of reflectors, nanocoating, and PCM with CuO nanoparticle. Experiments about the tray solar stills with flat and corrugated absorbers with a mixture of paraffin wax and CuO nanoparticles were conducted by Abdullah et al. (2021). The results showed that the accumulated freshwater production was 180% higher when compared to the conventional system. Also, the cost per litre was 0.028 $/l for the conventional still and 0.025 $/l for the modified still. Kumar et al. (2021) conducted experimental tests on three single-slope single-basin passive solar stills having identical dimensions and materials. The first one was a conventional one, the second was incorporated with paraffin wax, and the third was incorporated with silica nano-PCM. The results concluded that the incorporation of PCM and n-PCM improved the freshwater production by 51.22% and 67.07%, respectively. A hemispherical concentrator coupled with an active solar still was studied by Kumar et al. (2013). Cooper balls with paraffin wax inside were fixed on the absorber of the solar still. The results showed that there was a 26% enhancement in the daily freshwater production. Kabeel et al. (2016) coupled a double-pass solar air heater, alongside with a single basin solar still (with paraffin wax under the basin). The results showed that freshwater production increased by 108% when compared to the freshwater production of the conventional still at the same conditions. A single-slope solar still incorporated with a paraffin wax and a parabolic solar concentrator fixed under the solar still were performed by Kabeel et al. (2017). The experimental results showed that the daily freshwater production of the system was higher in the summer by a range of 55–65% and in the winter by a range of 35–45% when compared to the conventional one. Kabeel and Abdelgaied (2017) experimentally coupled a cylindrical parabolic concentrator (with a focal pipe) alongside with a solar still equipped (with an oil heat exchanger and 17.5 kg of paraffin wax). The freshwater production was compared to the conventional solar still under the same conditions. The results concluded that the freshwater production of the developed system was 140.4% higher than that of the conventional still. Khairat et al. (2020) integrated a parabolic trough collector (PTC) and a heat exchanger serpentine alongside with an under basin phase change material with a single-slope solar still. The results concluded that the daily production of freshwater for the conventional solar still at flow rates of oil of 1.5, 1.0, and 0.5 l/min and at flow rate of nano-oil of 0.5 l/min were 3.182, 4.67, 6.21, 8.79, and 11.14 l/m^2^.day, respectively. The efficiencies of the system at the said flow rates were 28%, 13.7%, 18%, 26%, and 34%, respectively. Sharma et al. (2022a, b) experimentally investigated a single-slope evacuated tubular collector (ETC) solar energy-based with water purifier (SEBWP) using N identical ETCs. The system’s performance parameters had been assessed using MATLAB, and the results had been then verified against experimental data. Theoretical and experimental values have been found to be reasonably in an agreement. The glass temperature, the water temperature, and the freshwater production were found to have correlation coefficient values of 0.9932, 0.9928, and 0.9951, respectively.

Danduprolu et al. (2022) presented a comprehensive review of the importance of the CFD tool in solar still analysis, performance estimation, and design improvements. Various approaches’ assumptions and governing equations had been presented. The findings revealed that the relatively simpler CFD modeling of only the humid air zone in the solar still, which is dependent on the availability of experimental data, has now evolved to an advanced level and can give predictive estimates using only the ambient atmospheric conditions and solar irradiation as input.

Negi et al. (2021) tried to synthesize the global trends and methods that have been tested in terms of integrating latent heat storage materials in solar stills. The review concluded that the solar still coupled with parabolic concentrator collectors along with the spiral tubes could be possible way in order to enhance the productivity of the combined system.

The effect of dissimilarity of mass flow rate and number of collectors on exergo-enviro-economic parameters for solar still of single slope type integrated with multi similar photovoltaic thermal flat plate collectors having series connection had been investigated analytically using MATLAB code for computing the different parameters by Sharma et al. (2022a, b). The results showed that the optimum number of thermal flat collectors for given value of mass flow rate of water had been found to be 10 from exergoeconomic parameter viewpoint and 6 from productivity viewpoint.

Singh et al. (2018) enhanced the exergoeconomic and enviroeconomic parameters for single-slope solar stills by incorporating *N* identical partially covered photovoltaic thermal (PVT) collectors. Three cases had been discussed: (i) single slope solar still incorporating *N* identical partially covered PVT flat plate collectors (FPC) (N-PVT-FPC-SS), (ii) single slope solar still incorporating *N* identical partially covered PVT compound parabolic concentrator collectors (N-PVT-CPC-SS), and (iii) conventional single slope solar still (CSSSS). The results showed that the kWh per unit cost based on exergoeconomic parameter is higher by 45.11% and 47.37%; environmental cost is higher by 65.74% and 90.02%; however, the output per unit input based on productivity is higher by 12.09% and lower by 26.83% for N-PVT-FPC-SS than N-PVT-CPC-SS and CSSSS, respectively.

Ahmed et al. (2022) presented modelling and experiments to improve the productivity of the solar still modified by a corrugated absorber plate and phase change material (PCM). The MATLAB model was used in a parametric study to optimize parameters such as glass cover thickness to reach to the maximum freshwater production. According to the results, the solar still with the PCM produced 4.5 l/day of freshwater at a cost of 42.34 USD/m^3^. In contrary, the solar still without PCM produced 4.1 l/day of freshwater at a cost of 43.6 USD/m^3^. The experimental data was also compared to the predictions of the MATLAB mathematical model, and there was a good agreement. The mathematical model results also showed that a solar still with a glass thickness of 4 mm was more productive than that with a glass thickness of 5 mm and 6 mm.

Alawee et al. (2022) conducted experimental tests on a conventional solar still (CSS) and a modified solar still (MSS). To boost the freshwater production of MSS, a copper water heating coil, an external condenser, and nanophase change material (PCM-Ag) were used. According to the findings, using a PCM and using an external condenser increased the freshwater production of MSS with heating coil by approximately 35% and 44%, respectively. Distilled freshwater costs 0.029, 0.024, and 0.022 $/l for the CSS, MSS-PCM, and MSS-EC, respectively.

A detailed comparison between single-basin and stacked solar still configurations based on thermodynamic and economic analysis had been conducted by Murugan et al. (2022). A single-slope single-basin and a double-basin solar still of same base area of 0.5 m^2^ were fabricated and tested. The results concluded that the average of the freshwater production of the single-basin and stacked still were 1.416 l/day and 1.913 l/day, respectively. The cost per litre of distilled water produced from a single-basin solar still was 29.9% higher than the stacked solar still.

Suraparaju and Natarajan (2021) designed and developed a novel bottom finned (solid and hollow) absorber basin in order to improve the heat transfer among the absorber and the phase change material. They compared the obtained results with conventional solar still results. The three single-slope solar stills was (i) conventional solar still (CSS), (ii) solar still with a hollow finned absorber inserted in energy storage (SSHFES), and (iii) solar still with a solid finned absorber inserted in energy storage (SSSFES). The productivity of the SSHFES was 4085 ml/m^2^.day, whereas the productivity of SSSFES and CSS was 3485 ml/m^2^ day and 2885 ml/m^2^ day, respectively.

From the previous review, it can be deduced that using PCMs is a good method for improving the freshwater production of the solar still especially at night time. Among other types of PCMs, paraffin wax was the most commonly used in all types of solar stills. Several methods for increasing solar freshwater production using various types of PCMs were investigated. The main concern of this study is to improve the performance of a single-slope conventional solar still by integrating a moderate power electric heater powered by PV solar modules. The solar heater was immersed inside the PCM storage (paraffin wax type) which is located below the solar still basin. Another concern of this study is investigating the impact of heating the paraffin wax under different operating temperature conditions. Also, the aim of fixing the electric heater inside the paraffin wax is to solve the problem of low thermal conductivities in most of the PCMs in general and make the solar still produce freshwater into the later hours of night. Unlike previous researches that improved the solar still’s performance only by using PCM, PCM with nanoparticles, or even small electric heaters that heated only the water inside the solar still, the method proposed in this research is considered a new method for improving the solar still's performance.

## Experimental setup

**Fig. 1:**
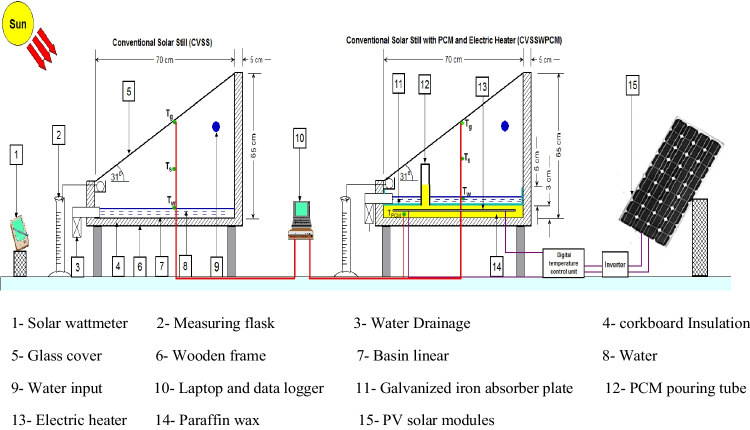
Schematic diagram of the experimental setup

**Fig. 2:**
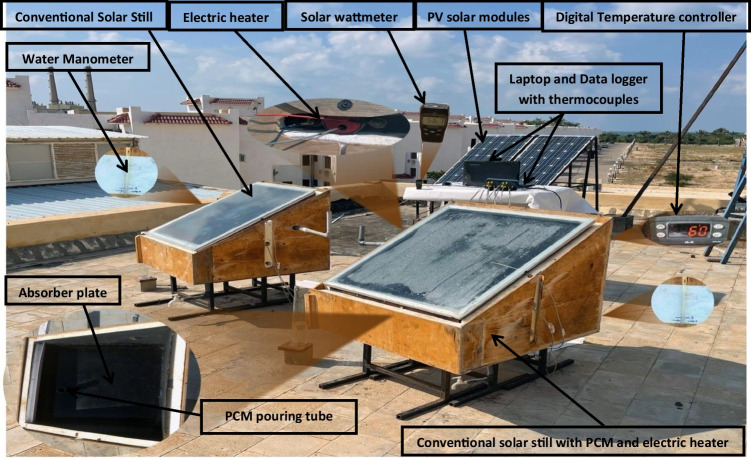
Photograph of the experimental setup

The solar stills under scrutiny were designed, installed, and tested in the Energy Laboratory, Department of Mechanical Engineering, Faculty of Engineering, Sinai University. The experimental work was performed over several days for each month during the spring and the summer of 2021 in Al-Arish city (latitude 31°N and longitude 33°E) in Egypt. The schematic diagram of the experimental setup is shown in Fig. 1, while Fig. 2 presents a photograph of the experimental setup. The experimental work consists of two solar stills: the first is a conventional solar still (CVSS), and the second is a conventional solar still with PCM and an electric heater (CVSSWPCM). The two solar stills have the same dimensions and are made of the same materials. The enhanced solar still was accomplished by a paraffin wax and an electric heater powered by PV solar modules.

The two stills are made of black wrought iron 2-mm-thick sheets that are mounted on a wooden frame. The sides and the base of the solar still are painted black to increase the solar absorptivity. The two stills have the same square basin area of 71.5 × 70 cm^2^ (about 0.5 m^2^). The elevations of the high-side and low-side walls of each still have been kept at 65 cm and 23 cm, respectively. The two stills have two 4-mm-thick transparent commercial glass covers, both have the same inclination angle. A 5-cm corkboard insulation material is used to reduce heat loss from the two stills to the ambient. The two solar stills are positioned on the east–west axis and fixed in the south direction. The slope of the condensing glass for each still is adapted to be equal to the latitude of the place (Al-Arish city) in order to accumulate the maximum amount of incident solar intensity. Three holes are made in each still: one for drainage, one for distilled water output, and the last one for feeding water into the still. With a 10-degree downward angle, a U-channel is welded along the inner sides of the low-side wall of each solar still. This downward inclination makes it easy for the condensate water to accumulate and glide through the U-channels before being collected in a calibrated flask. An airtight rubber gasket is used as a sealing between the solar still edges and the glass cover to prevent any vapour escape. In each solar still, four K-type thermocouples are connected to measure the temperatures of the water (*T*_*w*_) and the glass (*T*_*g*_); the average value is then recorded. The temperature sensors are connected to a data logger to record the temperatures on an hourly basis. Regardless of the insulation layer and the glass cover, the solar still (CVSSWPCM) is enhanced by adding a PCM reservoir, a 250-W electric heater, and an absorber plate coated with black paint. Located inside the modified still, the absorber plate is a 0.4-mm-thick galvanized iron sheet whose edges are 6 cm high.

**Fig. 3:**
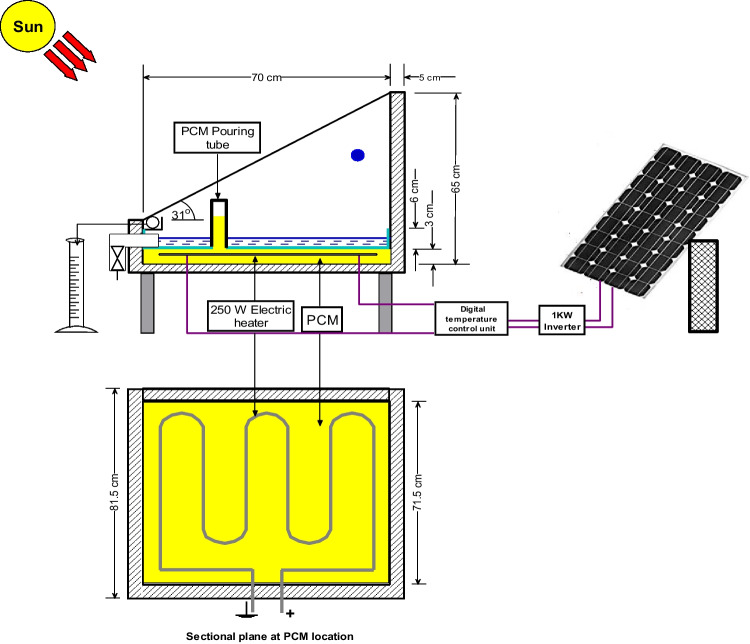
Schematic diagram of the enhanced solar still (sectional side and plane view)

**Fig. 4:**
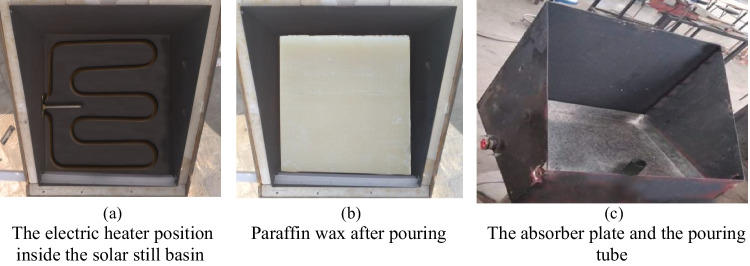
Illustrative photos of the CVSSWPCM

A 3-cm high PCM reservoir is installed inside the solar still (beneath the absorber plate) and filled with paraffin wax. Basing on the volume of the PCM reservoir, the used mass of paraffin wax is about 12 kg, weighted by a calibrated balance. It is mentioned that the volume of the paraffin wax is predicted to expand by 13% of its volume due to its change from a solid phase to a liquid phase. As a result, extra 1.5 kg of paraffin wax is added to ensure that the paraffin wax and the under absorber liner are firmly conjoined. Therefore, a total weight of the wax (13.5 kg) is used to fill the PCM reservoir. Paraffin wax is chosen as a PCM material owing to its low cost, non-toxicity, large latent heat of fusion, uniform melting, safety, and reliability. The thermo-physical characteristics of the paraffin wax employed in this study are shown in Table 1. A 12.5-mm wide hole is drilled in the absorber plate in order to vertically install a galvanized iron tube with a height of 30 cm. This tube is fixed in the hole using a spot welding process, rubber gasket, and heat resistant silicon. This tube is used to pour the melted wax into the storage tank. It acts as a vent used to accommodate the increased volume that results from the expansion of the paraffin wax during the melting process. It also acts as an opening through which the air bubbles (that emerged in the wax during the melting process) emit. Figure 3 shows a schematic drawing of the enhanced solar still. Figure 4 illustrates each part of the modified solar still individually.

**Table 1:** Paraffin wax thermo-physical properties (Haji-Sheikh et al. 1982)

Properties	Values
Melting point temperature	58 °C
Thermal conductivity	0.24 W/m °C
Liquid/solid heat capacity	2.51/2.95 kJ/kg °C
Liquid/solid density	760/818 kg/m^3^
Latent heat	226 kJ/kg

The electric heater is installed in the centre of the PCM reservoir and is powered by PV solar cells that are appropriate for the power of the electric heater used. The characteristics of the PV solar cells are shown in Table 2. A temperature controller is fixed between the input of the electric heater and the output of the PV solar cells to control the operating temperature of the paraffin wax. A single-phase high-frequency inverter (type PV181012 VPM) with a rated power of 1 kW is used in the experimental work to convert DC into AC to operate the heater. The PV system consists also of two solar batteries (model SG 1000H, 12 V, 100 AH, 20-hour rate) with a capacity of 93Ah (@25 °C).

**Table 2:** The PV solar module characteristics

Properties	Values
Cell type	Multi crystalline (156 × 156 mm)
No. of cells	72 cells (6 × 12)
Dimensions	1956 × 992 × 46 mm
Module type	TSM‒280PA14
Maximum power	280 W
Maximum power current	7.78 A
Maximum power voltage	36 V

## Experimental procedure

The experiments are conducted for several days during the spring and the summer of 2021. Different parameters are measured during the experiments: the solar intensity, ambient temperature, wind velocity, average glass cover temperature, average basin water temperature, PCM temperature, and accumulated output freshwater production. All parameters are recorded and tabulated every hour at a constant depth of saline water (2 cm) for both CVSS and CVSSWPCM and heater. All experimental measurements are taken to evaluate the performance of the two stills under the climate conditions of Al-Arish, Egypt. All experiments are conducted in convergent days during April 2021 and June 2021 to ensure that the solar intensity and the wind velocity variation do not change much during the reading days; taking the measurements in convergent days aims at decreasing the effect of the solar intensity and the wind velocity variation on the results during the comparison. The experiments show the crucial effect of using the paraffin wax as a heat storage medium in the presence of a working solar electric heater.

When heating the paraffin wax with the electric heater at the beginning of the experiment in the day light, the heat is stored in the paraffin wax as a sensible heat. The paraffin wax temperature reaches the melting point in one case study and exceeds it in others where it completely melts. The heat is then absorbed again in the form of sensible heat. In the night and the periods of a low solar radiation, the paraffin wax provides a sufficient source of heat for the basin water. So, using a paraffin wax and a solar electric heater guarantees that the solar still freshwater production continues until midnight during the experimental days.

## Error analysis

Instrument selection, condition, calibration, environment, observation, reading, and test planning can all lead to errors and uncertainties in the experiments. It is very important to estimate the accuracy of the measured and calculated parameters in order to make a correct analysis of the experimental results. The measured parameters include temperatures, solar radiation, wind velocity, and freshwater production, while the calculated parameters include instantaneous efficiency for the two solar stills. The Holman method (Holman 1994) was used to estimate the uncertainty in the experimental setup. The measurement uncertainty is defined as the root sum square of the instrumentation’s fixed error and the random error detected during multiple measurements. The degree of uncertainty in the results is estimated as follows:

1 $${W}_{R}=\sqrt{{(\frac{\partial \mathrm{R}}{\partial \mathrm{X}1}{W}_{1})}^{2}+{(\frac{\partial \mathrm{R}}{\partial \mathrm{X}2}{W}_{2})}^{2}+\dots +{(\frac{\partial \mathrm{R}}{\partial \mathrm{Xn}}{W}_{n})}^{2}}$$

where $W$_1_, $W$_2_, $W$_3_, …, $W$_*n*_ are the uncertainties in the independent variables. A set of measurements was done in order to measure “*n*” number of experimental variables. These measurements are used to calculate some desired results of the experiment. The solar still instantaneous efficiency can be calculated from the relation as follows,

2 $$\eta =\frac{{m}^{.}\times {h}_{fg}}{A\times I\left(t\right)+We}$$

where the average latent heat ${h}_{fg}$ is followed by Dashtban and Tabrizi (2011)

3 $${h}_{fg}={10}^{3}[25101.9-2.40706 {T}_{w}+1.92217\times {10}^{-3}{ T}_{w}^{2}-1.5863\times {10}^{-5} {T}_{w}^{3}$$

Table 3 shows the accuracy, range, and percentage error for various instruments used. It is clear from this table that all values are small compared to the data obtained and found to be within the allowable range of the measurements of the devices.

**Table 3:** Accuracy and error for various measuring instruments

No	Instrument	Accuracy	Range	% error
1	Thermometer	± 1 °C	0–100 °C	1
2	Thermocouple	± 0.1 °C	− 270–1820 °C	0.005
3	Solar watt meter	± 1 W/m^2^	0–2500 W/m^2^	0.04
4	Anemometer	± 0.1 m/s	0–12 m/s	0.83
5	Measuring flask	± 10 ml	0–1000 ml	1

Accordingly, the resulting errors of the calculated amount of daily freshwater production and in daily efficiency are ± 1% and ± 1.008%, respectively.

The thermodynamics law is used to examine the exergy balance. The exergy analysis determines the connection between the produced exergy and the total exergy input into solar desalination using (Shoeibi et al. 2022):

4 $${\eta }_{\mathrm{ex}}=\frac{{Ex}_{\mathrm{out}}}{{Ex}_{\mathrm{in}}}$$

The solar desalination exergy product is calculated as follows (Shoeibi et al. 2022):

5 $${Ex}_{\mathrm{in}}=\left(A \times \mathrm{ I}\left(t\right) \times \left(1-\frac{4{T}_{a}}{3{T}_{S}}\right)+\frac{1}{3}{\left(\frac{{T}_{a}}{{T}_{s}}\right)}^{4}\right)+We$$

where *T*_*s*_ shows the temperature of the sun (equal to 5727 °C). The exergy product of the solar still could be calculated through:

6 $${Ex}_{\mathrm{out}}=\left(\frac{{\dot{m}}_{\mathrm{ev}}}{3600}\times {h}_{fg}\times \left(1-\frac{{T}_{a}}{{T}_{w}}\right)\right)$$

## Results and discussion

The present study examines the effect of adjusting the operating paraffin wax temperature on the hourly and daily production of freshwater during the spring and the summer.

The experiments are carried out across several days in each month of the spring and the summer. The impact of using a solar-powered electric heater with the modified solar still to manage and stabilize the needed paraffin wax temperature is tested in four scenarios: (i) without an electric heater (only paraffin wax), (ii) at the temperature of 58 °C, (iii) of 60 °C, and (iv) of 65 °C. Under the same climatic conditions in both the spring or the summer, the outcomes are compared to the conventional solar still operating at the same climatic and the same design conditions.

**Fig. 5:**
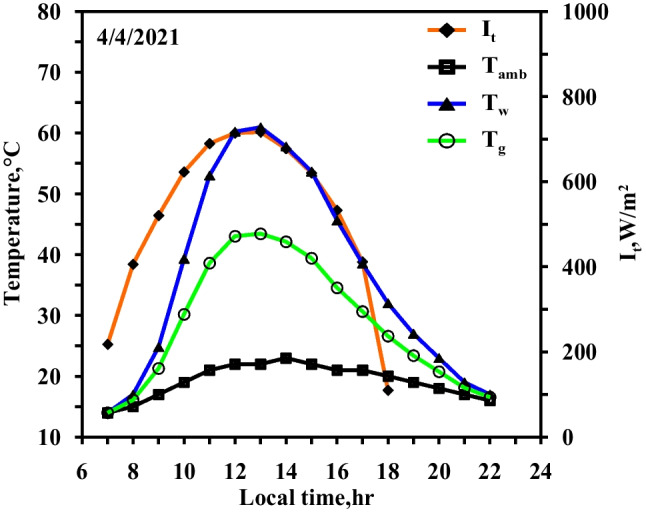
Hourly solar radiation and temperature variation for conventional solar still in spring

Figure 5 demonstrates the variation (i) in the temperatures of water and glass of CVSS, (ii) the ambient temperature, and (iii) the solar intensity with local time (all in case 1). During the spring experiments, the maximum solar intensity at noon period reaches 730 W/m^2^, while the sunlight period is 11 h. The temperatures of the water basin and the glass range from 14 to 60.8 °C and from 13 to 43 °C, respectively. With the passage of the daylight hours and the increase of the solar intensity reaching its maximum value, the temperatures of the water basin and the glass also increase until they reach their maximum values; the temperature differential between the water basin and the glass reaches the maximum value of 17 °C. The figure also shows that the ambient temperature varies along the day hours and reaches a maximum value at noon time. The average value of the ambient temperature on the day of the experiment is 16 °C.

**Fig. 6:**
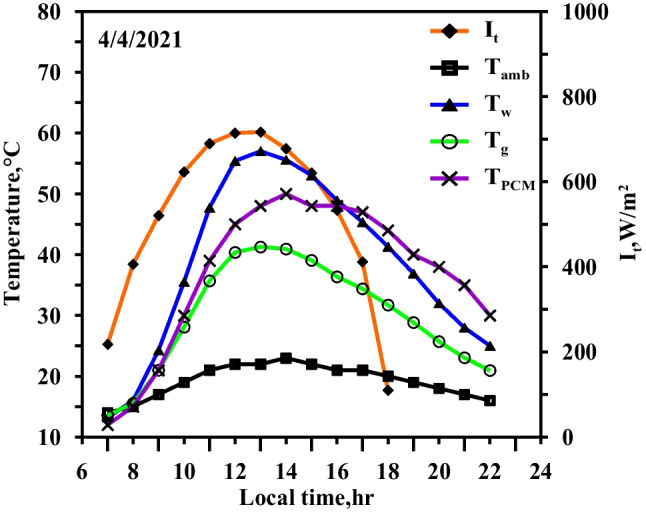
Hourly solar radiation and temperature variation for conventional solar still with PCM in spring

Figure 6 depicts the impact of using the paraffin wax without a heater in the modified solar still. The experimental results showed in this figure are obtained on the same day of case1 (Fig. 5) to demonstrate the effectiveness of using the paraffin wax (case 2). The figure also depicts the hourly change of the temperatures of the water, the glass, and the paraffin wax, as well as the solar intensity. In comparison to the typical situation, the water temperature in the case of using the paraffin wax is lower, specifically from 9:00 to 15:00 (charging period when a heat gain from solar energy was partially transmitted to the paraffin wax). Because the paraffin wax in the modified still does not reach the melting point, the water temperatures of the modified and the conventional stills simultaneously reach their maximum values (55 °C and 60 °C, respectively). The water temperature in the modified still is higher than that of the conventional still throughout the period from 15:00 to 22:00 according to the local time (discharging period). This is attributed to the fact that the heat stored in the paraffin wax, in the case of the modified still, is transferred to the water. Water temperature in the modified still reaches 9.9 °C at 19:00 as the paraffin wax discharges the commenced heat at 16:30. As a result, the production time of the modified still extends through the night time.

**Fig. 7:**
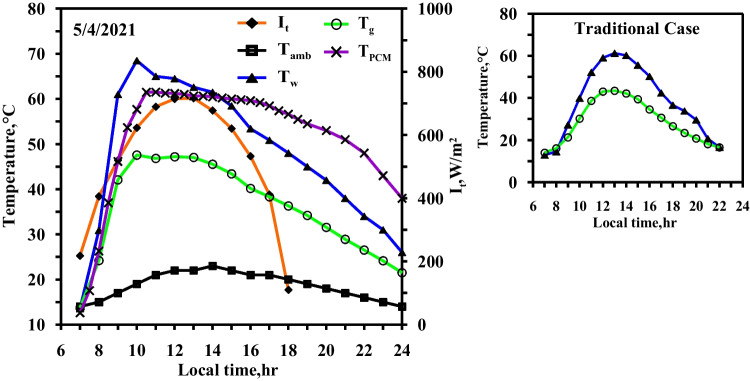
Hourly solar radiation and temperature variation for conventional solar still with PCM with heater at control temperature 58 °C in spring

Figure 7 displays the effect of using the electric heater to control the operating temperature of the paraffin wax at 58 °C (case 3). On the right side, Fig. 7 represents the temperatures of water and glass of the conventional solar still to compere between them, where the measurement is made on the same day. The electric heater starts in working from 7:30 to 16:30, depending on the availability of the electrical energy produced by the solar PV modules. It takes about two and a half hours to reach the operating temperature of 58 °C for the paraffin wax. From this figure, it can be seen that the water and the glass temperatures reach their maximum values approximately at the same time the paraffin wax reaches its maximum value (i.e., 58 °C).

The maximum temperatures of water, glass, and paraffin wax at 10:00 are 68 °C, 46 °C, and 60 °C, respectively. After 15 min (10:15), the temperature of the paraffin wax decreased again to reach to 58 °C since the stable condition is achieved after the digital temperature controller disconnects the electric heater from the power. From 10:15 to 16:30, the temperature of the paraffin wax is approximately constant. If the temperature of paraffin wax reduced by one degree, the digital temperature controller again connects the electric heater to the power in order to raise the temperature of the paraffin wax to the operating temperature (58 °C). The melting point of the paraffin wax is 58 °C. As for the paraffin wax to reach from its temperature at the beginning of the experiment (13 °C) to its melting point, the electric heater gives it heat (sensible heat). The electric heater, then, continues to give the paraffin wax heat (latent heat), while the wax’s temperature remains 58 °C.

The discharge of heat by the paraffin wax starts at 16:30 in two successive stages. Stage 1 occurs during the period from 16:30 to 19:30, when the temperature of paraffin wax decrease from 58 to 54 °C in the form of a sensible heat. Stage 2 occurs during the period from 19:30 to 24:00, when the paraffin wax temperature dropped from the melting temperature as the latent heat and sensible heat were expelled. Also, Fig. 7 shows that the water temperature’s curve rises rapidly between 7:40 and 10:00 owing to the continuous operating of the heater in order for the paraffin wax to reach the operating temperature. The heat transmission from the heater is likewise transmitted to the water. The electric heater functions only to maintain the temperature of the paraffin wax (at 58 °C). Once the electric heater achieves the desired temperature, the temperature of the water gradually declines. An amount of the water’s heat (which it gains from the heater) transfers to the paraffin wax, while another amount contributes in raising the evaporation rate of the water. The discharge of heat by the paraffin wax, which begins at 16:30 and ends at 24:00, causes the water temperature in modified still to be higher than that in the conventional still. The highest value difference in water temperatures of the two stills occur at 22:00 by the value of 17.4 °C. Also, it can be seen from Fig. 7 that the glass temperature of the modified still is not largely affected from the glass temperature of the conventional one since the two glasses of the two solar stills are exposed to the same wind speed and the same ambient conditions.

**Fig. 8:**
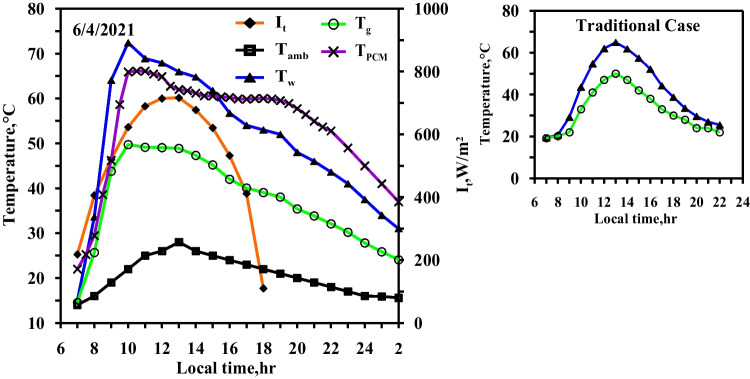
Hourly solar radiation and temperature variation for conventional solar still with PCM with heater at control temperature 60 °C in spring

Figure 8 displays the effect of using the electric heater to control the temperature of paraffin wax at 60 °C (case 4). It is clear that, from Fig. 8, the two curves of the water temperature and the paraffin wax are the same as those in case 3. The water temperature in (case 4) is higher than in the conventional still (case 1). The two maximum peaks of the curve occur at 9:00 and 21:00, and their values are 34 °C and 18.9 °C, respectively. The increase in the required temperature of the paraffin wax from 58 to 60 °C results in two extra hour extension for the fresh water production period on the next day. The delay of the discharge of heat by the paraffin wax affects the water temperature, and therefore, the condensation process was effective through the night and the beginning of the next day. The figure shows that the maximum temperatures of the water, the glass, and the paraffin wax are recorded approximately at 10:00 with values of 72 °C, 48 °C, and 65 °C, respectively. The paraffin wax temperature returns to the operating temperature of 60 °C after two and half hours approximately from disconnecting the electric heater. Also, the figure shows no remarkable difference in the glass temperatures of both of the solar stills.

**Fig. 9:**
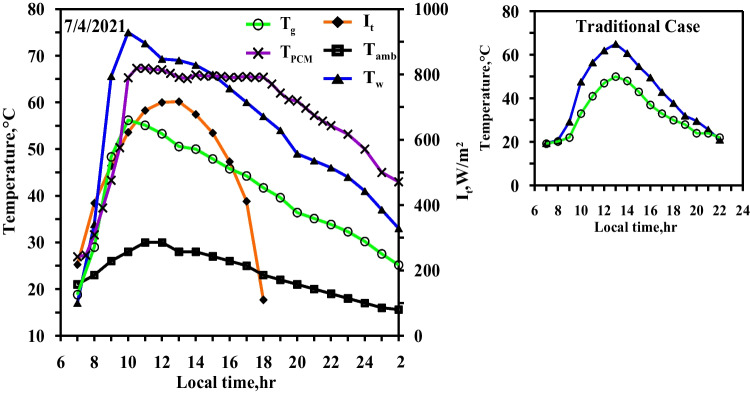
Hourly solar radiation and temperature variation for conventional solar still with PCM with heater at control temperature 65 °C in spring

Figure 9 shows case 5, where the required operating temperature of the paraffin wax is 65 °C, 7 degrees higher than the melting point. The paraffin wax discharges sensible heat from 19:00 to 22:00, resulting in an increase in water temperature over the temperature of conventional still, with a maximum difference of 25 °C during that time. The heat discharge from the paraffin wax to the water inside the modified solar still is extended to 4 h. The water temperature is higher than 50 °C for 11 h, which leads to a higher evaporation rate in the daylight periods. Also keeping the water temperatures at higher values that long period leads to higher evaporation rates in the night besides that, the condensation rates will increases through the night because of the low temperatures of glass and ambient in the night. The paraffin wax effect reaches extra 2 h in the next day as same condition of case 4.

The effect of varying the temperature of the paraffin wax during the summer is studied. The effect of integrating the paraffin wax with the solar still is more significant in the summer, as the daily solar intensity is higher by 25% than in the spring, which leads to the effectiveness of the use of the paraffin wax.

**Fig. 10:**
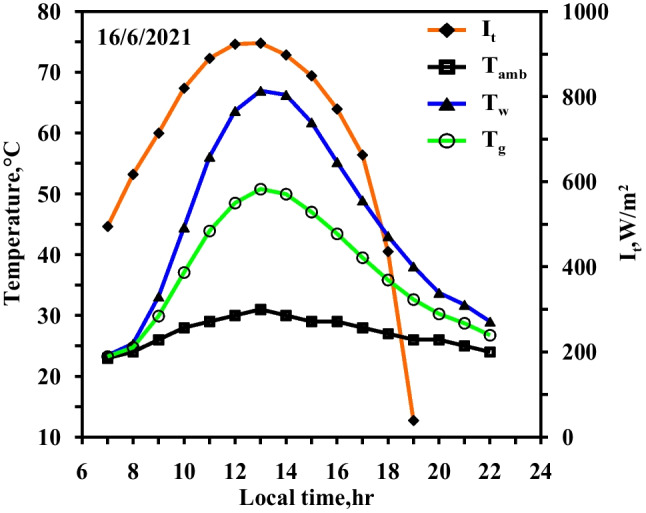
Hourly solar radiation and temperature variation for conventional solar still in summer

**Fig. 11:**
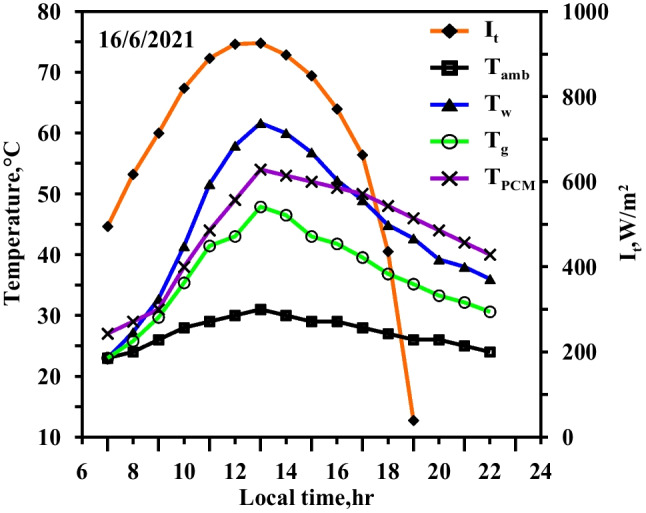
Hourly solar radiation and temperature variation for conventional solar still with PCM in summer

The same experiments of the spring days were done in the summer days with the same steps and same studied cases to confirm the obtained results from the spring days. The same curve trend behaviours are obtained but with values higher than of that obtained in the spring days because of the high ambient condition and the high solar intensity obtained in the summer days. Figures 10 and 11 show the effect of using the paraffin wax on the water and the glass temperatures compared to the conventional still (Fig. 10) at the same day. The paraffin wax temperature starts to increase sharply as the paraffin wax charge period starts from the beginning of the day until 13:00, reaching the melting point and then the paraffin wax gained the latent heat. The maximum values for the water and the glass temperature are 67 °C and 51 °C for the conventional solar still (case 1), respectively, and 61 °C and 48 °C for the paraffin wax case, as a portion of the solar radiation fallen on the solar still plate directed to the paraffin wax resulted in a decrease in water temperature in case (2) compared to the case (1) (conventional still case). Also, Figs. 10 and 11 show the variation of the solar intensity with local time. The solar intensity reaches its high value at 12:00 local time with a value of 950 W/m^2^.

**Fig. 12:**
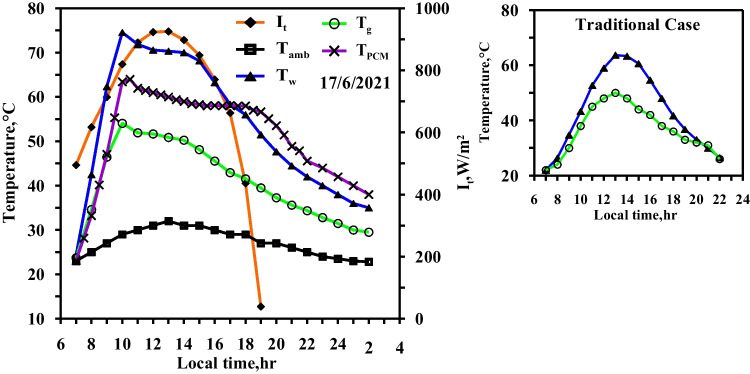
Hourly solar radiation and temperature variation for conventional solar still with PCM with heater at control temperature 58 °C in summer

**Fig. 13:**
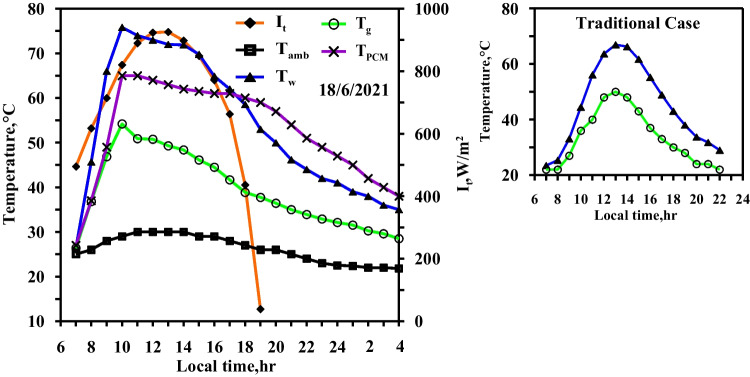
Hourly solar radiation and temperature variation for conventional solar still with PCM with heater at control temperature 60 °C in summer

**Fig. 14:**
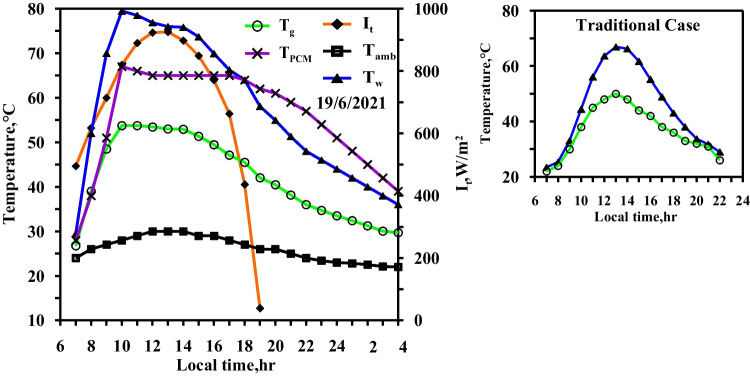
Hourly solar radiation and temperature variation for conventional solar still with PCM with heater at control temperature 65 °C in summer

Figures 12, 13, 14 show the hourly variation of the water, the glass, and the ambient temperatures during the summer for the three cases (cases 3, 4, and 5) at the three required paraffin wax temperatures. The paraffin wax curve follows the same behaviour as in the spring, except that the solar energy in the case of the summer allowed us to operate the heater more hours in the daylight because the daylight period is more than the night period in the summer days and thus maintains the temperature of the paraffin wax constant at its studied different operating temperatures till 19:30, Therefore, during the period 19:30 to 21:30, the paraffin wax releases sensible heat during discharge. The paraffin wax discharge period in the summer is longer than in the spring, lasting until the next day.

**Fig. 15:**
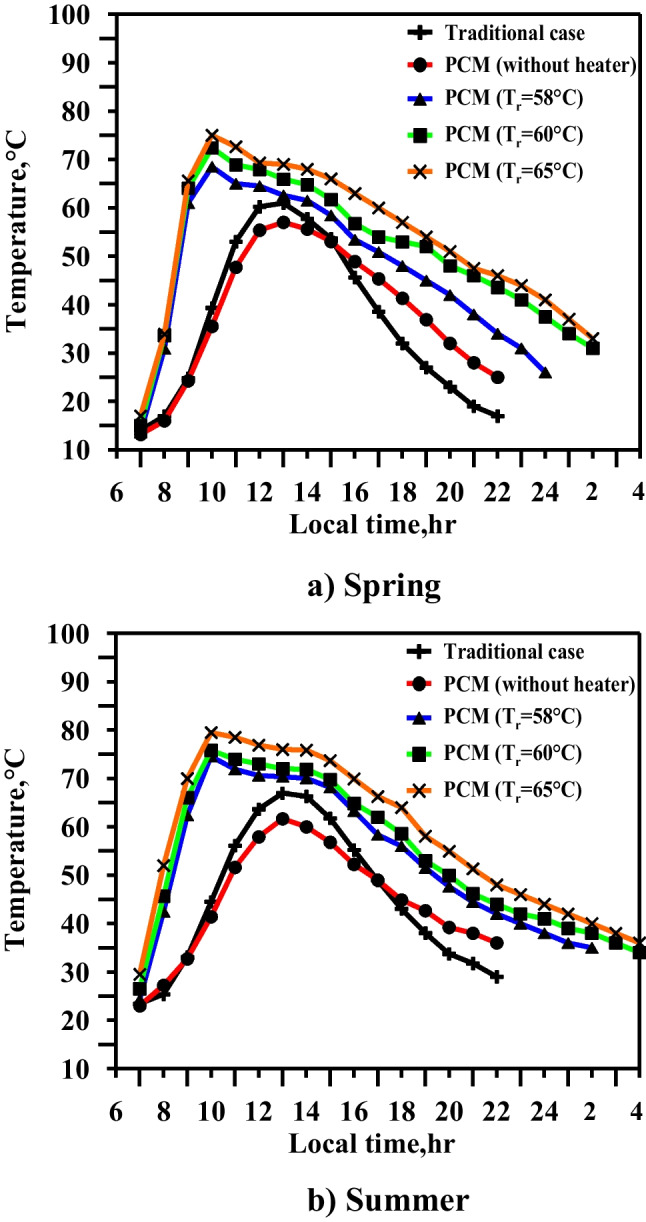
Water temperature for five cases in spring and summer

Figure 15 summarizes the effect of using the heater with paraffin wax at three studied operating temperatures, which leads to heating the water to high temperatures of up to 60 °C for a period of 6, 6, and 8 h in spring and 7, 8, and 9 h in summer.

**Fig. 16:**
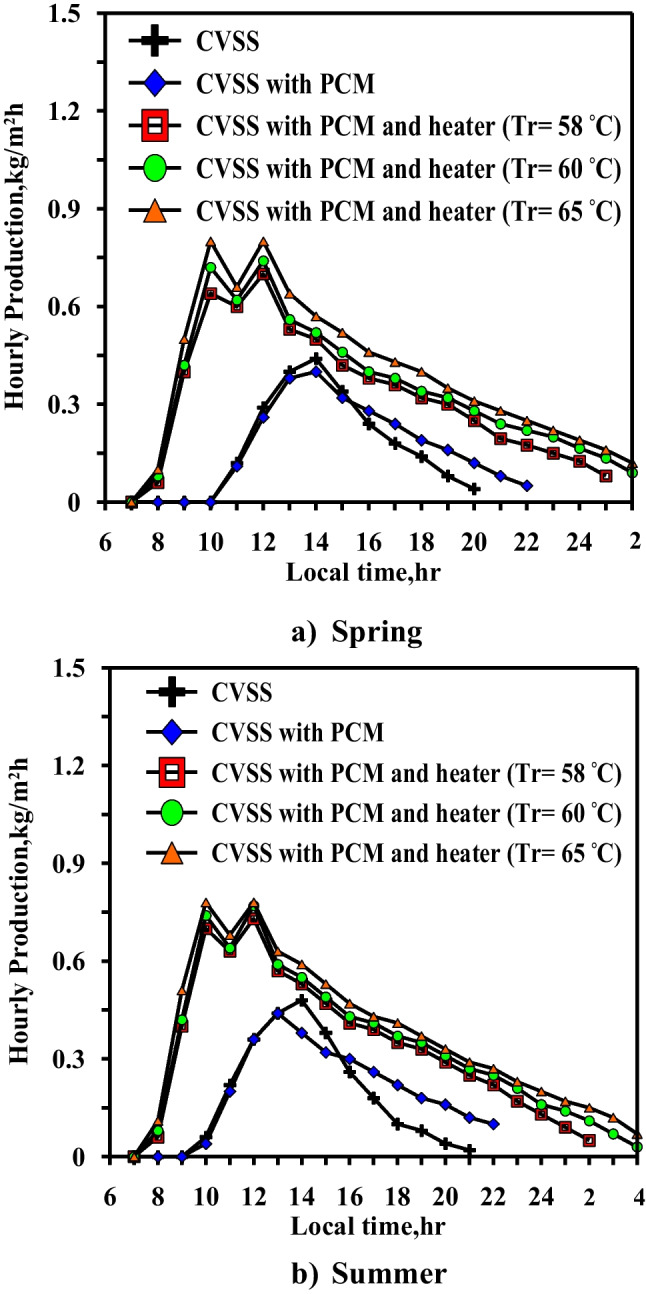
Hourly freshwater production for five cases

Figure 16 shows the effect of changing the hourly freshwater production during the spring and the summer days with local time. In the spring, the conventional case (case 1) shows that the freshwater production starts from 11:00 and ends approximately at 20:00 at the spring days with a higher value occurs at noon time of 0.43 kg/m^2^ h, while in the summer days, the freshwater production starts at 10:00 and ends at 21:00 with high production rate occurs at noon time with a value of 0.45 kg/m^2^ h. In case 2 (the still with the paraffin wax and without a heater), the freshwater production extends 2 h later in the spring days due to the storage effect of the paraffin wax. The maximum hourly freshwater production is 0.42 and 0.44 kg/m^2^ h at the spring and the summer days, respectively. The effect of changing the required paraffin wax temperature at the three values (58 °C, 60 °C, and 65 °C) is significant and affected the hourly freshwater production. The freshwater production starts at 8:00 and extends to 17 h in the case of (*T*_*r*_ = 58 °C) and 18 h for two cases (*T*_*r*_ = 60 °C and 65 °C) at the spring days. While at the summer days, the fresh water production starts at 8:00 local time and extends to 18 h in case of (*T*_*r*_ = 58 °C) and to 20 h for the other two cases. The maximum freshwater production for the three cases for *T*_*r*_ = 58 °C, 60 °C, and 65 °C is 0.7, 0.74, and 0.8 kg/m^2^ h, respectively. For the three cases, the curve of the hourly freshwater production has two peaks during the period from 10:00 to 12:00 local time. The first peak is related to the water temperature rise, as shown in Fig. 15. As the water temperature is higher than the paraffin wax temperature, it leads to the occurrence of amount of the heat transfer from the water to the paraffin wax to maintain the paraffin wax at the required temperature from 10:00 to 11:00. The other amount of the heat transfer in to high evaporation rate through this period. The water temperature increases again due to the solar intensity, causing the second peak in the hourly freshwater production which is slightly lower than the first peak or equal to the first peak in some studied cases. In the summer, the hourly freshwater production curve follows the same manner as in the spring, with the exception of the extension of the hourly freshwater production to 2 h later due to the higher solar intensity in the summer than in the spring.

**Fig. 17:**
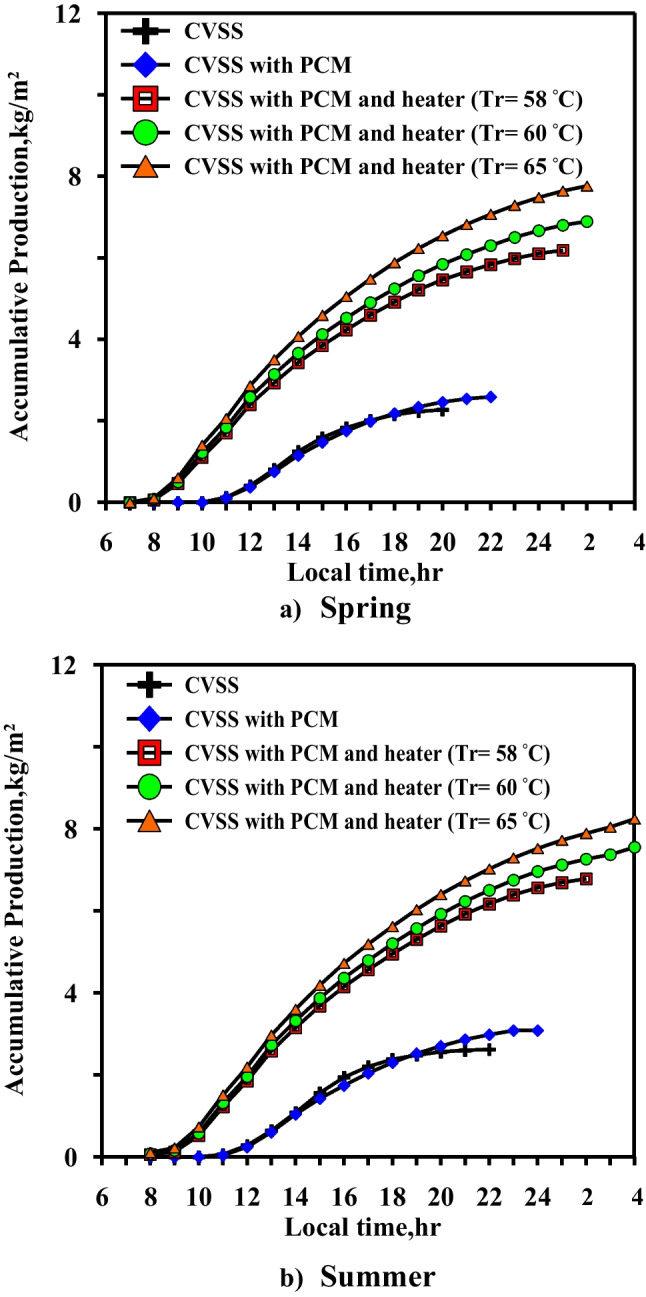
Daily freshwater production for five cases at **a** spring and **b** summer

**Fig. 18:**
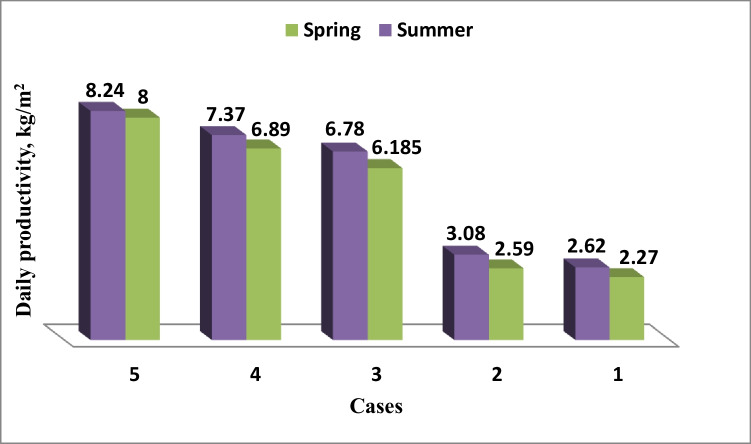
Comparison for the daily freshwater productivity for five cases in spring and summer

Figures 17 and 18 show the accumulative daily freshwater production for the five cases in the spring and summer, respectively. The figure shows that accumulative daily freshwater production increases with the increase of the local time. The accumulative daily freshwater production increased in the spring compared to the case of paraffin wax without the heater by 2.38, 2.66, and 3.1 times for the three paraffin wax required temperatures (58 °C, 60 °C, and 65 °C), respectively, while in the summer, it was 2.2, 2.39, and 2.67 times compared to the case of paraffin wax without the heater. Case 5 (paraffin wax temperature, *T*_*r*_ = 65 °C) has the highest daily freshwater production either in the spring or in the summer.

**Fig. 19:**
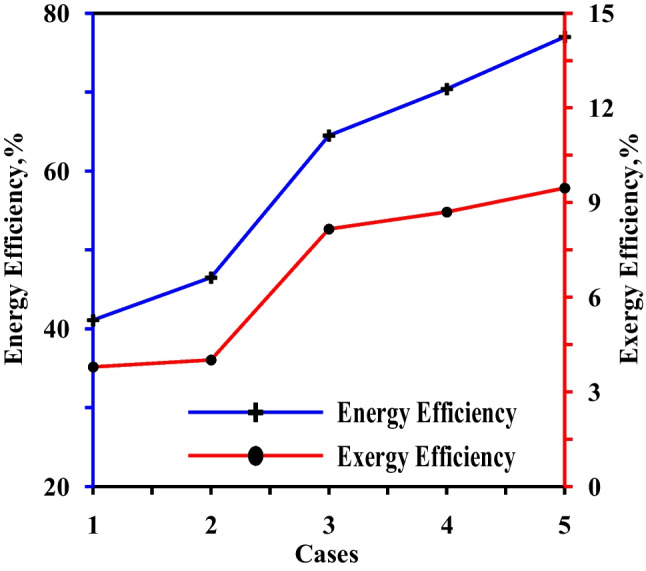
The average energy and exergy efficiencies for five cases

Figure 19 depicts the five solar still scenarios’ average daily energy efficiency and exergy. As shown, the instance (5) has the maximum energy efficiency since it has the largest daily freshwater production. The findings revealed that the exergy efficiency in instance (5) was the greatest, owing to greater production.

Table 4 shows the average annual fresh water production for the solar stills; the conventional solar still and the modified solar still under different studied operating temperatures. The table shows case 5 for the CVSSWPM and electric heater with operating temperature of 65 °C with average value of 2842 l/m^2^ year.

**Table 4:** The average annual fresh water production of the studied five cases in the present study

Case	Case(1) CVSS	Case(2) CVSSWPCM	Case(3) CVSSWPCM (*T*_*r*_ = 58 °C)	Case(4) CVSSWPCM (*T*_*r*_ = 60 °C)	Case(5) CVSSWPCM (*T*_*r*_ = 65 °C)
Average freshwater production per year (l/m^2^ year)	855.8	992.3	2268.9	2495.5	2842

### Cost analysis

To assess the economics of the modified solar still, many methodologies were utilized, taking into account the key elements influencing the cost of purified water, such as site, fed water quality, system capacity, and component pricing (Chaichan and Kazem 2015), to measure the economics of the modified solar still. Table 5 shows the price of system components in the Egyptian market for conventional and modified solar stills. According to the Egyptian Solar Atlas, the number of cloudy and rainy days [Nc] in Sinai would never exceed fifteen days. Operational days were considered to be 350 days each year.

**Table 5:** Charge of components per one m^2^ of solar still absorber area

	CSS		Proposed solar still	
Component	Quantity	Total cost ($)	Quantity	Total cost ($)
Solar still	1	75	1	75
Modules of solar panel (280 W)	-	-	2	100
Inverter	-	-	1	20
Heater (250 W)			1	5
Paraffin wax			13.5 kg	30
Σsum		75		230

The techniques were the capital recovery factor (CRF) and the annual/annual/fixed/cost (FAC) factor. The values were calculated as in Table 5. The salvage value of the complete apparatus after its lifetime (*N* = 10) was set at 20% of the total fixed cost. Table 6 displays the CPL for the typical solar still and the modified scenario (5). The conventional solar still had an estimated CPL of 0.0180 $/l/m^2^, whereas the modified scenario (5) had an estimated CPL of 0.0176 $/l/m^2^.

**Table 6:** Economic analysis results

Calculated parameters	Equation	CVSS	Proposed solar still (Case 5)
Life, *n*		20	20
Interest per year *i*, %		12	12
Capital cost (CS), $		75	232
Salvage value (S), $	*S* = 0.2 × *CS*	15	47
(CRF)	$$CRF= \frac{i {(1+i)}^{n}}{{(1+i)}^{n}-1}$$	0.177	0.177
(SFF)	$$SFF=\frac{i}{{\left(i+1\right)}^{n}-1}$$	0.057	0.057
Fixed annual cost (FAC), $	$$FAC=CRF \times CS$$	13.275	41.7595
AMC, $	*AMC* = 0.15 × *FAC*	1.991	12.5279
Annual salvage value (ASV), $	$$ASV=SFF \times S$$	0.162	2.7119
TAC, $	*TAC* = *FAC* + *AMC* − *ASV*	16.46	51.5755
Annual freshwater production, l		917	2884
Cost per one litre of distilled water freshwater production, CPL, $/l	*CPL* = $$\frac{TAC}{ L}$$	0.0180	0.0176

Table 7 compares the outcomes of the current investigation to those indicated by previous studies. The findings are consistent with the literature.

**Table 7:** Comparisons between present study and different solar still studies

Ref	Type of solar still	Max yearly yield (l/m^2^)	Cost per litre ($/l/m^2^)
Present study	Conventional solar still with PCM and electric heater powered by PV module	2884	0.0176
Kabeel and Abdelgaied (2016)	Passive conventional single slope solar still with paraffin wax as PCM	2210	0.03
Rufuss et al. (2018)	Passive conventional single slope solar still with paraffin wax and CuO nanoparticles	660	0.026468
Yousef and Hassan (2019)	Passive conventional single slope solar still with paraffin wax and steel wool fibres as porous medium	1345	0.050
Kabeel and Abdelgaied (2017)	Active single slope solar still with paraffin wax under basin + oil serpentine from parabolic solar concentrator	3298	0.0177
Khairat et al. (2020)	Active single slope solar still with parabolic solar collector and paraffin wax in the receiver evacuated tubes and in the still	3900	0.0154
Abdullah et al. (2021)	Active corrugated tray solar still with paraffin wax mixed with Cuo nanoparticles and electric heaters	2040	0.025
Prasad et al. (2022)	Solar still with photovoltaic modules and electrical heater	2230	0.028

#### Benefit cost ratio

One of the engineering economics methodologies for evaluating designs in terms of cost is the benefit cost ratio approach. The technique is a practical and well-known method for project appraisal that includes an economic study of private investment efforts. The benefit–cost ratio is calculated as follows (Kosmadakis et al. 2009):

7 $${\text{BCR}} \, \text{=} \, \text{UAB/TAC}$$

The following equation can be used to calculate the current value of benefit from solar desalination (Shoeibi et al. 2022):

8 $$\mathrm{UAB}=K\times \mathrm{POW}$$

POW is the price of water, which varies by country and has been as low as 0.28 $/l in Egypt. An investment must have a benefit-to-cost ratio greater than one in order to be more efficient.

#### Exergoeconomic constraint

The exergoeconomic constraint is established as financial analysis optimizing while factoring for the device’s exergy analysis, and it is produced using the following formula (khairat et al. 2022):

9 $${R}_{\mathrm{EN}}=\frac{{\left({E}_{\mathrm{en}}\right)}_{\mathrm{out}}}{\mathrm{TAC}}$$

10 $${R}_{\mathrm{EX}}=\frac{{\left({E}_{\mathrm{ex}}\right)}_{\mathrm{out}}}{\mathrm{TAC}}$$

#### Environmental parameter

All parameters were evaluated throughout this step depending on the quantity of CO_2_ contamination decreased by the solar still. However, the majority of the components of a solar sill, such as iron sheets, aluminium sheets, plexiglass, and pipes, are powered by fossil-fuel-generated energy, which is harmful to the environment. A large number of very harmful and destructive chemicals are discharged into the environment during the manufacture of these parts (Rajaseenivasan and Srithar 2016). CO_2_ elimination and CO_2_ emissions are used to compute environmental economic parameters.

#### ***CO***_***2***_*** emissions***

The cost of CO_2_ generation and delivery per kWh is now around 0.96 kg (Sovacool 2008). Furthermore, the CO_2_ emissions per kWh are equal to 2 kg due to transmission (20%) and distribution (40%) losses due to faulty equipment. Solar desalination’s yearly CO_2_ emissions and lifetime CO_2_ emissions are calculated based on:

11 $$\mathrm{ACDE}=\frac{2 \times {E}_{\mathrm{in}}}{N}$$

12 $$\mathrm{CDED}=2\times {E}_{\mathrm{in}}$$

#### ***CO***_***2***_*** reduction***

Solar desalination reduces CO_2_ emissions by around ((*E*_en_)_out_ × 2) for kilogram CO_2_ per year. As a consequence, the CO_2_ decrease during a lifetime may be written as ((*E*_en_)_out_ × 2 × *n*). To compute the net amount of CO_2_ reduction per ton throughout a lifetime, apply the following calculation (Shoeibi et al. 2022):

13 $$EP=\frac{2\left({\left({E}_{\mathrm{en}}\right)}_{\mathrm{out}}\times N-{E}_{\mathrm{in}}\right)}{1000}$$

#### Enviroeconomic parameter

The enviroeconomic parameter is defined as the cost of lowering CO_2_ emissions during the life of a solar desalination system. CO_2_ per ton is expected to cost around $14.5 USD/ton (CCT) (Khairat et al. 2022).

14 $$\mathrm{EPP}=\frac{2\left({\left({E}_{\mathrm{en}}\right)}_{\mathrm{out}} \times N-{E}_{\mathrm{in}}\right)}{1000}\times \mathrm{CCT}$$

#### Exergoenvironmental analysis

The exergoenvironmental parameter is determined as follows (Shoeibi et al. 2022) and examines carbon dioxide emission reduction based on exergy output in solar still:

15 $$\mathrm{EPx}=\frac{2\left({\left({E}_{\mathrm{ex}}\right)}_{\mathrm{out}} \times N-{E}_{\mathrm{in}}\right)}{1000}$$

#### Exergoenviroeconomic constraint

Exergoenviroeconomic investigation is a method for evaluating the cost of CO_2_ reduction while taking exergy into consideration.

16 $$\mathrm{EPPX}=\frac{2\left({\left({E}_{\mathrm{ex}}\right)}_{\mathrm{out}}\times N-{E}_{\mathrm{in}}\right)}{1000}\times \mathrm{CCT}$$

Table 8 displays the benefit cost ratio for each of the five scenarios with varying interest rates and durations. In each of the five scenarios, the benefit-to-cost ratio was greater than one. According to the findings, the benefit cost ratio in case (5) was roughly 18% greater than that in the traditional cases.

**Table 8:** Benefit cost ratio of solar stills

Solar still	*n*/years	*i*, %	TAC	POW/$	*K*	UAB/$	*B*/*C*
**CASE (1)**	20	0.12	11.338863	0.28	387.59	108.5252	9.57108303
	20	0.2	17.63	0.28	387.59	108.5252	6.15571185
	40	0.12	10.44	0.28	387.59	108.5252	10.3951341
	40	0.2	17.259	0.28	387.59	108.5252	6.28803523
**CASE (2)**	20	0.12	15.8744	0.28	528.22	147.9016	9.31698836
	20	0.2	24.68	0.28	528.22	147.9016	5.99277147
	40	0.12	14.62	0.28	528.22	147.9016	10.1163885
	40	0.2	24.16	0.28	528.22	147.9016	6.12175497
**CASE (3)**	20	0.12	34.77	0.28	1162.77	325.5756	9.36369284
	20	0.2	54.07	0.28	1162.77	325.5756	6.0213723
	40	0.12	32.02	0.28	1162.77	325.5756	10.1678826
	40	0.2	52.9	0.28	1162.77	325.5756	6.1545482
**CASE (4)**	20	0.12	34.77	0.28	1263.955	353.9074	10.1785275
	20	0.2	54.07	0.28	1263.955	353.9074	6.54535602
	40	0.12	32.02	0.28	1263.955	353.9074	11.0526983
	40	0.2	52.9	0.28	1263.955	353.9074	6.69012098
**CASE (5)**	20	0.12	34.77	0.28	1413.16	395.6848	11.3800633
	20	0.2	54.07	0.28	1413.16	395.6848	7.31800999
	40	0.12	32.02	0.28	1413.16	395.6848	12.3574266
	40	0.2	52.9	0.28	1413.16	395.6848	7.47986389

Table 9 displays the embodied energy of the five scenarios. To generate distinct components, the conventional and case (5) used approximately 219.58 kWh and 1843 kWh of energy, respectively. The instance (3)–(5) solar still had nearly nine times more embodied energy than the typical one, according to the findings.

**Table 9:** Embodied energy of various components of solar desalinations (Shoeibi et al., 2022c)

Type of solar still	Name of component	Energy density		Mass of component/kg	Embodied energy/kWh
		MJ kg^−1^	kWh kg^−1^		
**Case (1)**	Glass	31.5	28.3	4	113.2
	Body	25	6.9	10	69
	Insulation	55.6	15.44	0.5	7.72
	Basin coating	90	25	0.5	12.5
	PVC pipe	77.2	21.4	0.2	4.28
	Support (galvanized)	50	13.9	3	41.7
	Rubber gasket	11.83	3.28	0.6	1.968
**Total embodied energy (kWh)**					**219.58**
**Case (2)**	Glass	31.5	28.3	4	113.2
	Body	25	6.9	10	69
	Insulation	55.6	15.44	0.5	7.72
	Basin coating	90	25	0.5	12.5
	PVC pipe	77.2	21.4	0.2	4.28
	Support (galvanized)	50	13.9	3	41.7
	Paraffin wax (PCM)	714	198.3		198.3
	Copper heater	100	27.7	2	55.4
**Total embodied energy (kWh)**					**460.55**
**Cases (3–5)**	Glass	31.5	28.3	4	113.2
	Body	25	6.9	10	69
	Insulation	55.6	15.44	0.5	7.72
	Basin coating	90	25	0.5	12.5
	PVC pipe	77.2	21.4	0.2	4.28
	Support (galvanized)	50	13.9	3	41.7
	Copper heater	100	27.7	2	55.4
	Photovoltaic panel	98,800		3 m^2^	1470
	Paraffin wax (PCM)	714	198.3	13.5	198.3
**Total embodied energy (kWh)**					**1843**

Table 10 displays the exergoeconomic parameter for various lives and interest rates, taking exergy and energy desalination into consideration. Because of the large yearly energy and exergy output and low freshwater production in scenario 2, the exergoeconomic parameter is lower in various states. Furthermore, when comparing the scenario 5 to the traditional one, the exergoeconomic gains in terms of energy and exergy are around 6.6% and 66%, respectively.

**Table 10:** Exergoeconomic parameter for solar stills

Solar still	*n*/year	*i*	TAC	Annual (*E*_en_)_out_/kWh	Annual (*E*_ex_)_out_/kWh	*R*_En_/kWh ^$−1^	*R*_Ex_/kWh $^−1^
**Case (1)**	20	0.12	11.34	665.03	57.31	58.65	5.05
	20	0.2	17.63	665.03	57.31	37.72	3.25
	40	0.12	10.44	665.03	57.31	63.70	5.49
	40	0.2	17.26	665.03	57.31	38.53	3.32
**Case (2)**	20	0.12	15.87	753.00	60.59	47.43	3.82
	20	0.2	24.68	753.00	60.59	30.51	2.46
	40	0.12	14.62	753.00	60.59	51.50	4.14
	40	0.2	24.16	753.00	60.59	31.17	2.51
**Case (3)**	20	0.12	34.77	1655.28	196.37	47.61	5.65
	20	0.2	54.07	1655.28	196.37	30.61	3.63
	40	0.12	32.02	1655.28	196.37	51.70	6.13
	40	0.2	52.9	1655.28	196.37	31.29	3.71
**Case (4)**	20	0.12	34.77	1807.12	209.88	51.97	6.04
	20	0.2	54.07	1807.12	209.88	33.42	3.88
	40	0.12	32.02	1807.12	209.88	56.44	6.55
	40	0.2	52.9	1807.12	209.88	34.16	3.97
**Case (5)**	20	0.12	34.77	2055.00	227.76	59.10	6.55
	20	0.2	54.07	2055.00	227.76	38.01	4.21
	40	0.12	32.02	2055.00	227.76	64.18	7.11
	40	0.2	52.9	2055.00	227.76	38.85	4.31

Table 11 shows the environmental and exergoenvironmental parameters for the five solar still scenarios throughout a 20-year lifespan. As can be seen, scenario (5) has a lower exergoenvironmental parameter than case (1). CO_2_ emissions were discovered to be reliant on energy and exergy generation over the system’s lifetime. Case (5) and traditional solar stills reduced carbon dioxide emissions by 166.4 and 28.72 tons, respectively, according to the environmental study. Also, the table shows the enviroeconomic parameters for cases (5) and (1) which were 2316.69 $ and 410.14 $, respectively. Also, the table shows that Scenario (5) and traditional solar stills have exergoenvironmental parameters of 148.3 $ and 20.73 $, respectively.

**Table 11:** Environmental and exergoenvironmental parameter of solar stills

	Case (1)	Case (2)	Case (3)	Case (4)	Case(5)
Lifetime, years	20	20	20	20	20
Embodied energy, kWh	217.112	3212.5	3298	3220.2	3305
Annual energy output, kWh	718	2326	2361	2783	4160
Annual exergy output, kWh	46.6	225	287	341	421
CO_2_ emission during life time, kg	434.224	6368	6368	6440.4	6610
CO_2_ reduction during lifetime, ton	28.72	46.52	46.52	46.52	166.4
Environmental parameter, ton CO_2_	28.28577	86.615	87.844	104.8796	159.79
Enviroeconomic parameter, $	410.142	1255.91	1273.74	1520.75	2316.96
Exergoenvironmental parameter, ton CO_2_	1.429776	2.575	4.884	7.1996	10.23
Exergoenviroeconomic parameter, $	20.73175	37.3375	70.818	104.3942	148.335

## Conclusion and remarks

Water shortage is considered as one of the biggest challenge that faces the world recently. One of the best solutions to overcome this challenge is to get potable water from the salty water by using solar stills. Different enhancement methods are used to increase the still freshwater production in many ways. In this work, the effect of integrating an electric heater powered by solar energy with a conventional single-slope solar still with the paraffin wax as PCM mounted under the solar still basin (CVSSWPCM) is studied and evaluated. The still’s performance is tested under the same climatic conditions of Al-Arish, Egypt, during the spring and summer months of 2021, and the results are compared with the conventional one. Several parameters are measured during the experiment days, and different cases are studied with and without using a heater operating at temperatures of 58 °C, 60 °C, and 65 °C. The obtained results showed the following:

- The accumulated productivity of CVSS is 2.27 and 2.62 l/m^2^ per day at spring and summer, respectively, and the CVSSWPCM enhanced the freshwater production by 14% and 17.5% at spring and summer, respectively.
- The accumulated productivity of CVSSWPCM is enhanced by 172.5% and 158.8% using heater at control temperature of 58 °C at spring and summer, respectively.
- The accumulated productivity of CVSSWPCM is enhanced by 252.4% and 214.5% with heater at control temperature of 65 °C at spring and summer, respectively.
- The economic evaluation of the proposed solar still is performed with the aid of cost per litre. The solar still, with a heater operating at 65 °C, has a higher exergoeconomic value than the conventional one.
- The maximum CO_2_ mitigation in the cases of (5) and traditional solar desalination is approximately 160 tons and 28 tons, respectively.
- Exergoenviroeconomic parameters are 20.7 $ for case (1), 37.3 $ for case (2), 70.8 $ for case (3), 104.4 $ for case (4), and 148.3 $ for case (5).

In order to enhance the solar still performance with a new technique, it should use multi-electric fans powered by the PV system and adding different nanoparticles to the proposed system for studying the effect of these modifications on solar still productivity.

## Data Availability

Not applicable.

## Symbols and subscripts

*A*: Surface area, m^2^
AC: Alternative current
AMC: Annual maintenance cost
ASV: Annual salvage value
CPL: Cost per litre, $/l
CVSS: Conventional solar still
CVSSWPCM: Conventional solar still with phase change material and electric heater
CRF: Capital recovery factor
DC: Direct current
*E*: Energy, W
*E*_in_: Embodied energy, kWh
*E*_*x*_: Exergy, W
EP: Environmental parameter, ton CO_2_
EP_X_: Exergoenvironmental parameter, ton CO_2_
EPP: Enviroeconomic parameter, $
EPP_X_: Exergoenviroeconomic parameter, $
FAC: Fixed annual cost
*h*_*fg*_: Latent heat, kJ/kg
*I*_*t*_: Solar intensity, W/m^2^
*i*: Interest rate, %
*K*: Average annual freshwater production, l
*m**˙*: Water production rate, l/s
*n*: Life time of the still, years
*N*_*c*_: Number of cloudy days
PCM: Phase change material
POW: Price of water, $
*S*: Salvage value, $
SFF: Sinking fund factor
TECs: Thermoelectric coolers
*T*_*a*_: Ambient temperature, K
*T*_*s*_: Solar temperature, K
*T*_*r*_: Operating temperature of the paraffin wax
*T*_*w*_: Water temperature, K
*UAB*: Present worth of benefit, $

## Subscripts

CO_2_: Carbon dioxide gas
